# PhotoId-Whale: Blue whale dorsal fin classification for mobile devices

**DOI:** 10.1371/journal.pone.0237570

**Published:** 2020-10-12

**Authors:** Rosa I. Ramos-Arredondo, Blanca E. Carvajal-Gámez, Diane Gendron, Francisco J. Gallegos-Funes, Dante Mújica-Vargas, J. B. Rosas-Fernández

**Affiliations:** 1 SEPI-Culhuacan, Instituto Politécnico Nacional, Ciudad de México, México; 2 SEPI-ESCOM, Instituto Politécnico Nacional, Ciudad de México, México; 3 CICIMAR, Instituto Politécnico Nacional, Ciudad de México, México; 4 SEPI-Zacatenco, Instituto Politécnico Nacional, Ciudad de México, México; 5 Departamento de Ciencias Computacionales, Tecnológico Nacional de México, Cuernavaca-Morelos, México; 6 Secretaría de Educación, Ciencia, Tecnología e Innovación, Ciudad de México, México; Institute of Deep-sea Science and Engineering, Chinese Academy of Sciences, CHINA

## Abstract

Photo-identification (photo-id) is a method used in field studies by biologists to monitor animals according to their density, movement patterns and behavior, with the aim of predicting and preventing ecological risks. However, these methods can introduce subjectivity when manually classifying an individual animal, creating uncertainty or inaccuracy in the data as a result of the human criteria involved. One of the main objectives in photo-id is to implement an automated mechanism that is free of biases, portable, and easy to use. The main aim of this work is to develop an autonomous and portable photo-id system through the optimization of image classification algorithms that have high statistical dependence, with the goal of classifying dorsal fin images of the blue whale through offline information processing on a mobile platform. The new proposed methodology is based on the Scale Invariant Feature Transform (SIFT) that, in conjunction with statistical discriminators such as the variance and the standard deviation, fits the extracted data and selects the closest pixels that comprise the edges of the dorsal fin of the blue whale. In this way, we ensure the elimination of the most common external factors that could affect the quality of the image, thus avoiding the elimination of relevant sections of the dorsal fin. The photo-id method presented in this work has been developed using blue whale images collected off the coast of Baja California Sur. The results shown have qualitatively and quantitatively validated the method in terms of its sensitivity, specificity and accuracy on the Jetson Tegra TK1 mobile platform. The solution optimizes classic SIFT, balancing the results obtained with the computational cost, provides a more economical form of processing and obtains a portable system that could be beneficial for field studies through mobile platforms, making it available to scientists, government and the general public.

## Introduction

The first documented investigations of pattern recognition for animals began with the bottlenose dolphin on the coast of the Gulf of Mexico by researcher David K. Caldwell in 1955 [1], but it was not until 1970 that the observation of cetaceans used this technique. There are research works that study photo identification. Currently, the photo-id method is used in a wide variety of studies such as those focused on life cycles, group structures, geographical characteristics, population structures, population size estimates, and migrations, among others [2–9]. In the case of cetaceans, the marks used for photo-id may be caused by parasites, predator attacks, conspecifics, anthropogenic activities and congenital conditions [10]. However, these marks can be modified over time; consequently, other characteristics have been considered as identification mechanisms. The dorsal fin contour or marks from injuries are some, which generally are among the most accurate features to use for photo-id studies as demonstrated in previous work [10]. Photo-id is a minimally invasive technique [11], which has the capacity to demonstrate the potential consequences of human impacts and/or management actions by quantifying the spatial relationships between populations and the variables that define or reflect their habitat preferences [12]. The application of photo-id and electronic tagging in field studies has shown a relatively high degree of site fidelity [13], which is useful in order to establish possible conservation strategies. Systems based on computer vision, which are focused on image classification, have long attracted the attention of image processing researchers because the results obtained form the basis of many environmental and socioeconomic applications [14]. However, the classification of images acquired in uncontrolled media through surveillance cameras remains a recurring topic of study due to many factors, such as the complexity of the landscape in the study area, the various performance characteristics of the camera, the size and movement of the subject, the framing, and the camera’s sensitivity, which can affect the success of the classification result [15–17]. Most photo-id procedures consist of three steps. The first step is the manual selection and trimming of an area of interest to the individual within the image, after which the person in charge of the photo-id classifies and catalogs the images according to their criteria or experience. The second step is an automated algorithmic comparison between the sample and an image library that rates candidates by their coincident probability. The final step is a visual comparison of sample-candidate pairs to confirm positive matches [18]. Photo-id software has been developed to facilitate the task of identifying a diverse set of species, such as in [19–31]. Each of these photo-id systems have been developed on a laptop and personal computer due to the complexity of the proposals developed, such as the work shown in [32] that compares four animal photo-id systems. In the case of the blue whale, an identifying characteristic is the shape of its dorsal fin observed on both the right and left flanks. The dorsal fin of the blue whale can be grouped according to its shape; this classification was proposed by Gendron et al. [33], who explained that photographs taken of the dorsal fin must be observed on both flanks. Because the photo-id systems referenced above requires the help of an expert to detect the characteristics points, the resulting classification may contain errors or a certain bias and, consequently, may vary for the same individual. To identify an animal, it is necessary to extract its main characteristic transforms. For example, the Scale Invariant Feature Transforms (SIFTs) are usually vectors with a large number of components, which can generate redundant information or contain similar points or very close values between them, with a high computational cost [34,35]. These points are extracted from the contour of the dorsal fin of the blue whale on both flanks. We propose the elimination of coincident characteristic points using statistical discriminators such as the variance, standard deviation, mode and cross-correlation. Then, the results will verify that the data that were not discriminated provide sufficient information to carry out the identification and classification of the shape of the dorsal fin, resulting in an economical, fast and feasible solution. Currently, there are different solutions for data classifiers, such as the fuzzy C-means (FCM) [36], the K-means [37], the cluster-based density estimation (DBSCAN) [38], and more complex classifiers, such as support vector machines [39]. In each of these classifiers, initial values are required to make pertinent adjustments for different variations of the data entered and identify their behavior. Depending on the complexity of the problem, dimensionality reduction can be applied to the acquired data; in the particular case presented in this study, this reduction is necessary because the data will be processed on a mobile platform. Therefore, it is necessary that the classification techniques be simplified, reliable, efficient and available during the photo-id process. Systems capable of identifying animals in their habitats are an interesting challenge for image processing researchers due to the ability to achieve correct identification in the shortest possible execution time, guaranteeing intuitive and reliable development. Currently, the photo-id of the blue whale is done visually, so it can take several hours in the laboratory to identify, catalog and store the results obtained during the sightings. Therefore, it is important to consider the following points. Who uses the results?, the CICIMAR-IPN scientist. What do CICIMAR-IPN scientists use it for? After identifying and cataloging blue whales in the field for conservation purposes, where are the results used? In field expeditions off the coast of Baja California Sur, Mexico, identification has been developed as a passive data collector [40]. Innovation in new technologies and the accessibility of sensors, social networks, hi-fi cameras, interoperability between various devices and platform, storage capabilities, cloud computing, and computing on mobile development platform all together are powerful tools that are currently available on mobile platform, as shown in [41]. They are available to any researcher, naturalist, biologist or the general public, and there are applications that help or collaborate on information collection [42]. The current contributions of people on various technological platforms using the Internet or the different available social networks create the citizen sensor network [43] that is derived from the concept of citizen science, which has been used in various areas of knowledge for joint collaboration between the government, scientists and volunteers interested in the subject for conservation and ecology purposes. In this study, we present the results obtained during the research and implementation of PhotoId-Whale for the photo-id of blue whales for classification using mobile platform. We propose a classifier for the characteristic data obtained by the optimization of the SIFT presented in this research that reduces the coincidence of the characteristics. We illustrate its possible implementation on mobile platform and obtain greater portability in field studies. In the case of this research, we validated the results on the Jetson Tegra TK1 mobile development platform, which has several advantages such as the following: low cost, low power consumption and high applicability [44,45].

## Methods and materials

### Median estimation classifier methodology

**Fig 1:**
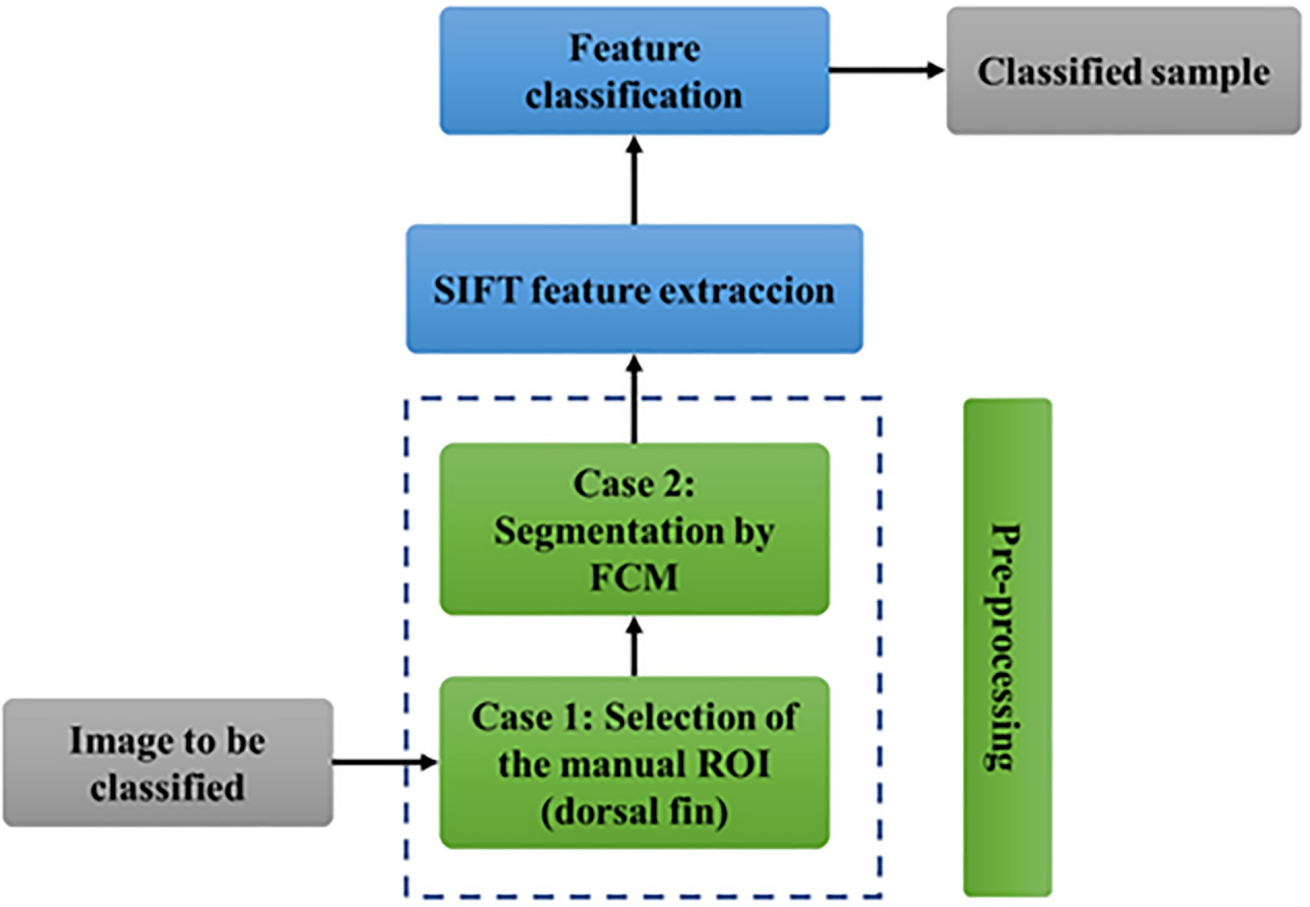
Image processing block diagram of the photoid-whale classification.

The methodology for the classification and identification of the dorsal fin of the blue whale is described in this section. A block diagram of the implemented stages is shown in Fig 1. From this figure, it can be seen that the first step is to obtain an image from the blue whale database. The second step is the preprocessing of the image, which is divided into two substages: i) the selection of the region of interest (ROI) and ii) the segmentation of the image to extract the portion to be classified. The third step is the extraction of the characteristics by means of the SIFT and of the results obtained from the elimination of the redundant data through determining the variance and covariance of the final vectors. The refined vector is immediately entered into the data classifier to obtain the dorsal fin of the blue whale, which is then classified; the results are subsequently stored in the database for monitoring.

Each of the previous steps is explained below in further detail.

Step 1. Obtain the image to be classified. The image is obtained from the database or acquired in the field.

Step 2. Image Pre-Processing: This stage is divided into two substages, which are described below.

**Fig 2:**
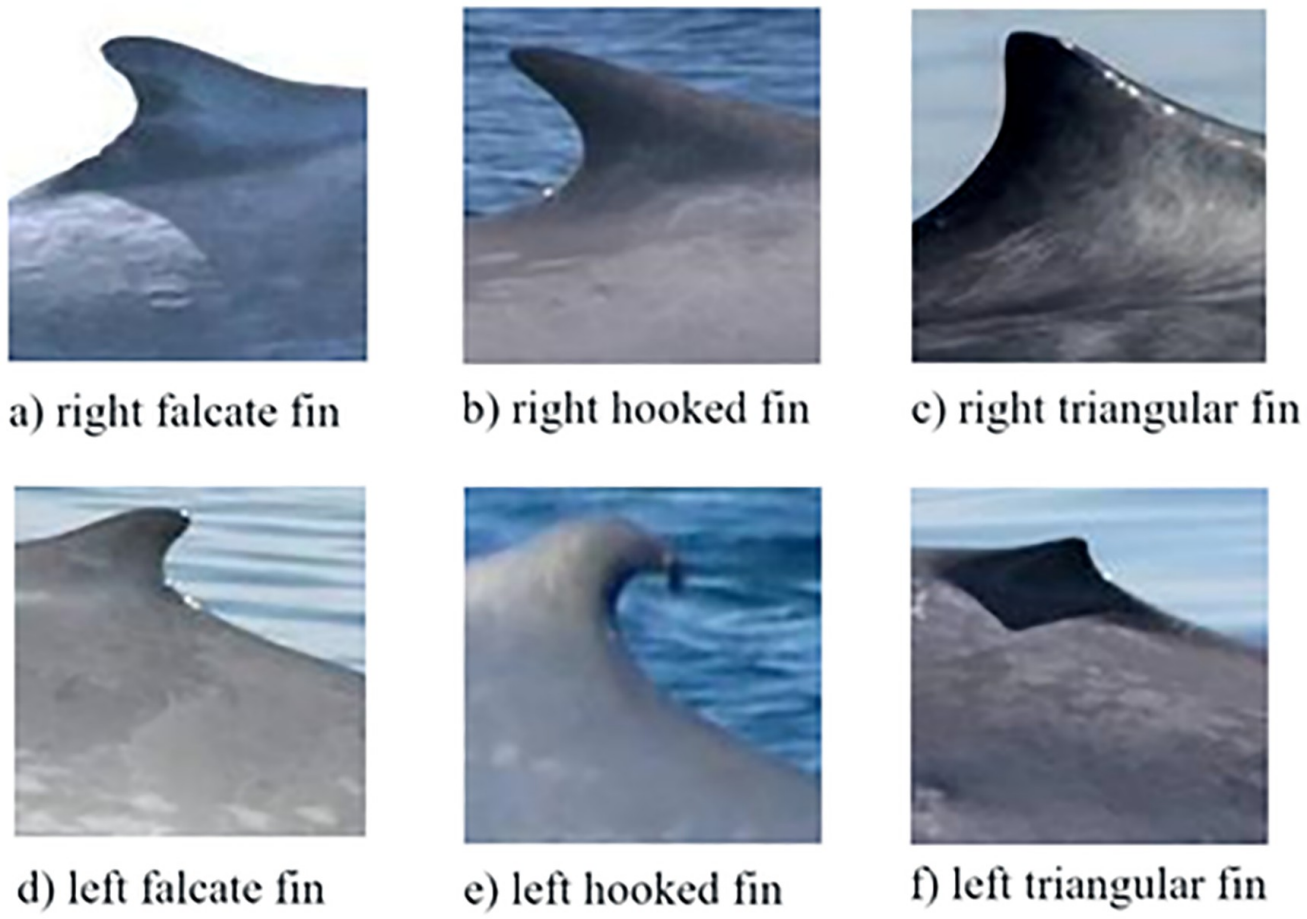
ROIs of different dorsal fins for the classification of the blue whale [33].
- Extraction of the dorsal fin from the image background: At this stage, the algorithm developed in reference [46] is implemented, which isolates the contour of the dorsal fin (Fig 3A) and eliminates existing noise in the ROI of the image (Fig 3B).

- ROI selection: By means of a graphical interface, the initial point is indicated, mainly in the area of the dorsal fin according to the criteria proposed Gendron et al. [33]. Example images of extracted ROIs are shown in Fig 2.

**Fig 3:**
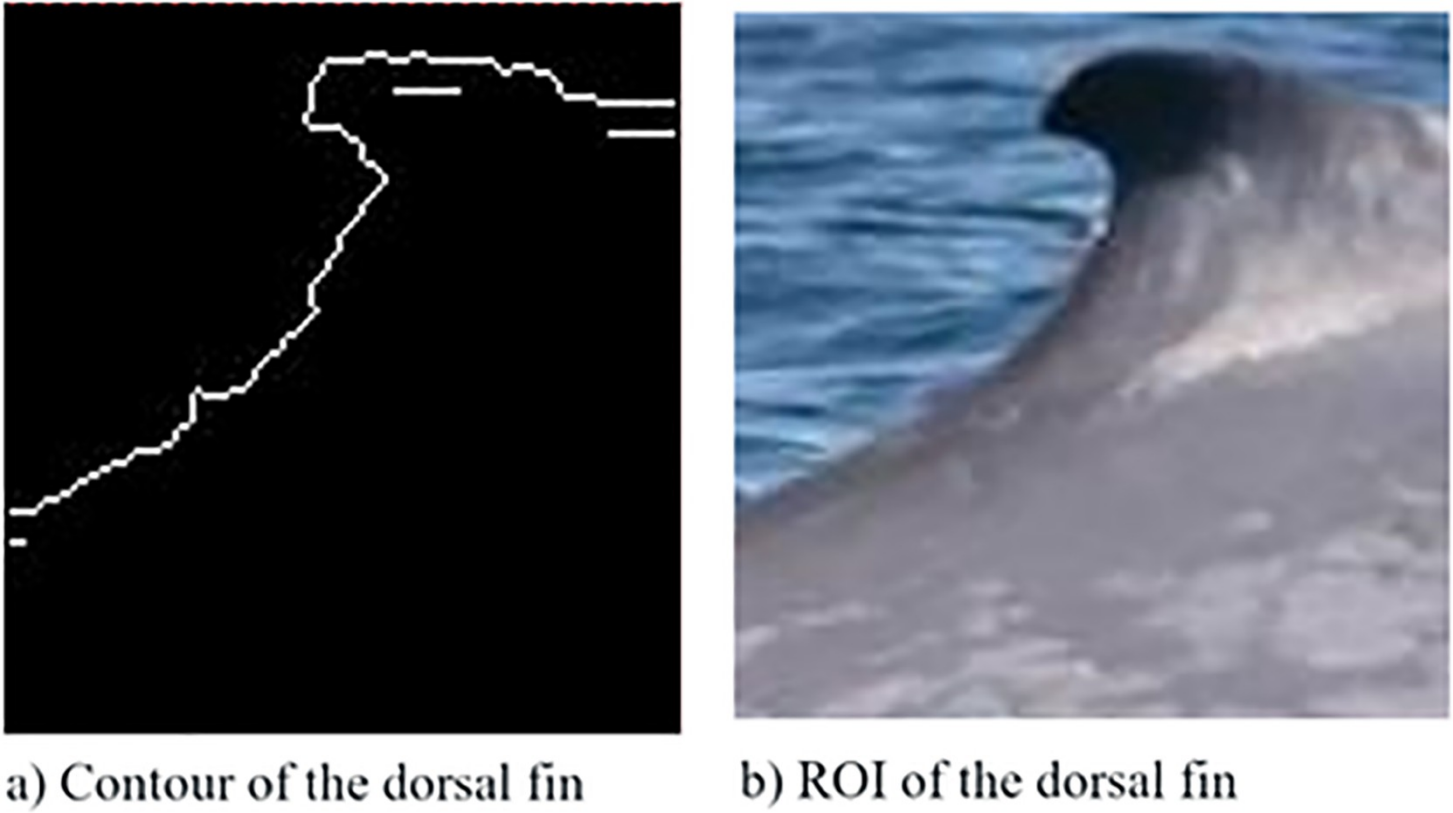
Dorsal fin of the blue whale: a) Contour of the segmented ROI, and b) Nonsegmented ROI.

From the extraction of the contour of the dorsal fin, data reduction is performed to subsequently proceed to step 3 on a mobile platform.

**Fig 4:**
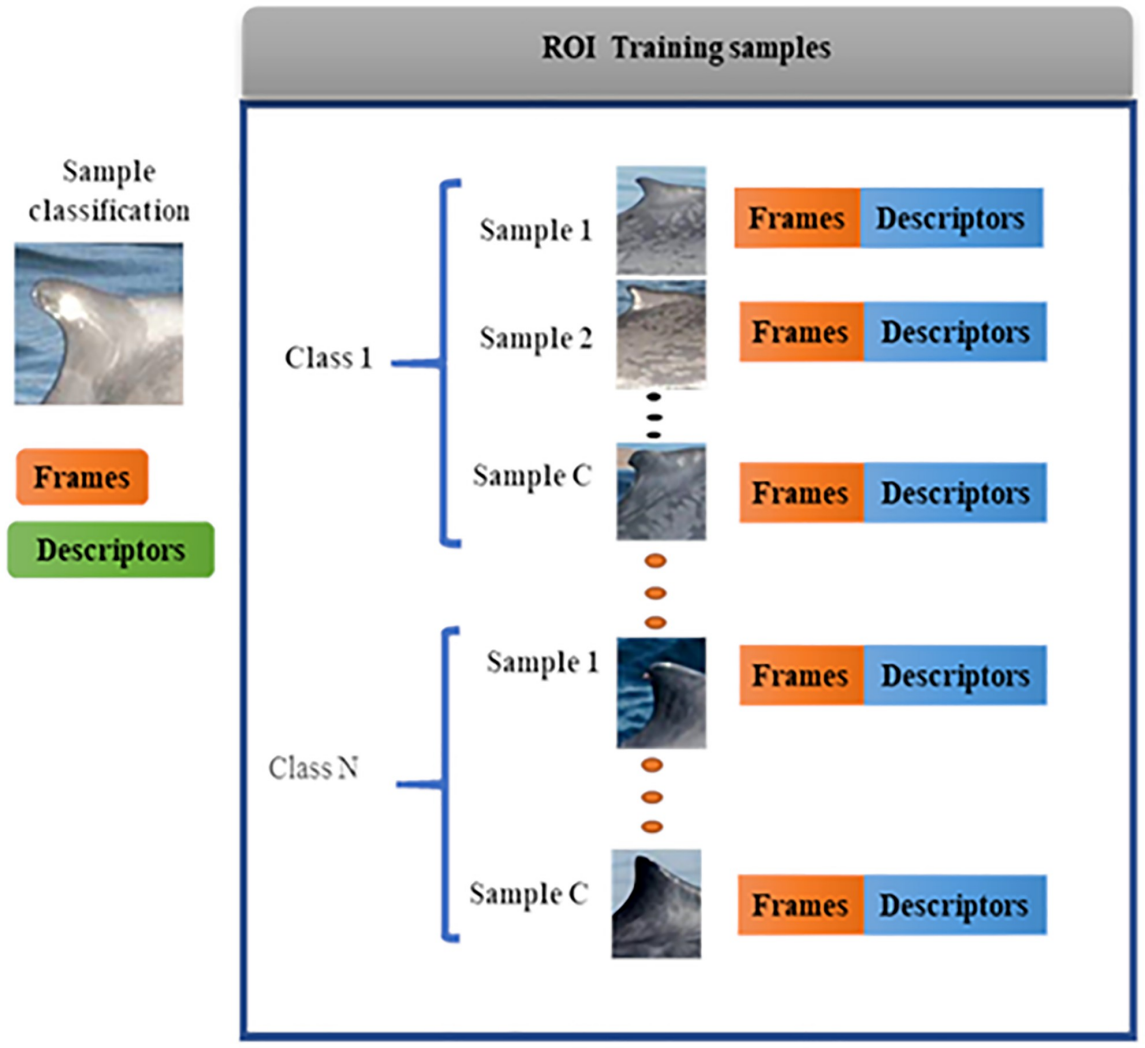
Block diagram of sample imaging training.

Step 3. Extraction of the main characteristics: From the result obtained in step 2, SIFT is applied to the contour of the dorsal fin. Fig 4 shows a block diagram of the considerations made in the extraction of the main characteristics of the processed images obtained in step 2.

Internally, for each class defined by Gendron et al. [33], the shape, size and orientation characteristics are extracted. According to the SIFT methodology [47], the first step is the detection of the scale from the contour of the dorsal fin, which is useful during the identification process because the obtained image may depend on the shooting distance. The formal description of this step is detailed below:

Scale detection: The scalar space *L (x*, *y)* of an image is obtained from the convolution of the input image I_ROI_ through a Gaussian filter *G (x*, *y*, *σ)* at different scales of σ [26,47], as shown in Eq 1:

$$L\left(x,\;y,\;\sigma \right)=\left(G\left(x,\;y,\;k\sigma \right)-G\left(x,\;y,\;\sigma \right)\right)*I_{ROI}\left(x,\;y,\;\sigma \right)$$ (1)

where $G(x,y,\sigma )=\frac{1}{2\pi \sigma^{2}}e^{\frac{-(x^{2}+y^{2})}{2\sigma^{2}}}$ represents a Gaussian filter, which is applied in both dimensions *(x*,*y)* of the I_ROI_ image plane.

To obtain the different scale versions of the I_ROI_ image, it is necessary to multiply σ with different constants k to obtain the projections of the contiguous scales (where k > 1). The original scale is subtracted from each of its projections, obtaining the differences from the original image *D*_*m*_, as shown in Eq 2:

$$D_{m}\left(x,\;y,\;\sigma \right)=L\left(x,\;y,\;k\sigma \right)-L\left(x,\;y,\;\sigma \right)$$ (2)

From the vector obtained in Eq 2, in order to eliminate redundant scales, the standard deviation is obtained and used as the threshold (Eq 3); this value helps to minimize the number of redundant samples in order to maximize the performance of resources on a mobile platform.

$$threshold=\sigma_{D_{m}}=\sqrt{\sum_{m=1}^{n}\left({\left(D_{m}\left(x,\;y,\;\sigma \right)-{\overset{-}{D}}_{m}\left(x,\;y,\;\sigma \right)\right)}^{2}\right)/n}$$ (3)

where *D*_*m*_ is the vector obtained from the scale differences (Eq 2), *m* represents the number of scales, $\bar{D}=\overset{n}{\underset{m=1}{-}}D_{m}/n$ is the average value of *D*_*m*_, and *n* is the total number of sample elements in the scale vector. This value will provide each of the thresholds required to obtain the significant points of the contour of the dorsal fin.

The new thresholding vector *D*_*new*_(*x*,*y*,*σ*) is obtained using the following equation:

$$D_{new}=D_{m}\left(x,\;y,\;\sigma \right)\leq \sigma_{D_{m}}$$ (4)

The next stage of the SIFT methodology involves locating the points of interest in the images, which serve as a reference to identify the unique characteristics of the dorsal fin. The points obtained constitute the differences among each of the contour shapes and are described below.

Points of interest: From the new scale vector, which is optimized by the scale selection threshold *D*_*new*_ to identify the characteristic points, it is necessary to locate where the values of the vector obtained in the previous stage increase or decrease. These vectors are useful due to the low contrast in the ROIs of the images of the dorsal fin since the fin can be confused with the sea.

The search for extreme values on the spatial scale produces multiple candidates. The points that are not selected are the low contrast ones since they are not stable to changes in lighting and noise. Eq 5 shows how the points of interest are located within the ROI contour, whose locations are given by references [26,47]:

$$z=-\frac{\partial^{2}D^{-1}{}_{new}(x,y,\sigma )}{\partial x^{2}}\frac{\partial D_{new}(x,y,\sigma )}{\partial x}$$ (5)

Subsequently, the vectors are arranged according to the orientation of the points obtained from Eq 5 as explained below.

Orientation mapping: This step assigns a constant orientation to the characteristic points based on the properties of the contour of the dorsal fin obtained in the previous steps. The characteristic point descriptor can be formed in relation to this orientation, resulting in invariance to rotation, which is important to highlight because images can be taken at different shooting angles. The procedure to find the orientation of the points is as follows [47]:

- Obtain the scalar values of the points of interest selected in Eq 5.
- Calculate the magnitude M:
  $$M(x,y)=\sqrt{\left((L\left(x+1,y\right)-{L\left(x-1,y\right))}^{2}+(L\left(x,y+1\right)-{L\left(x,y-1\right))}^{2}\right)}$$ (6)
- Calculate the orientation θ:
  $$\theta (x,y)={tan}^{-1}\left((L\left(x,y+1\right)-L\left(x,y-1\right))/(L\left(x+1,y\right)-L\left(x-1,y\right))\right)$$ (7)
- Locate the highest points of the histograms from the values obtained from Eqs 6 and 7. The characteristic points are obtained with the same orientation as that of the local values above 80%.

Finally, the particular descriptors of the characteristic points obtained in the previous steps must be identified, so they are arranged according to the following.

*Descriptors of the characteristic points*: To obtain the descriptors, the gradient of the vectors obtained from the selection criteria of the previous step is finally calculated. The vectors obtained from the gradient are rotated so that they are aligned with the point located on the ROI to be labeled with a Gaussian scale value, *σ*_*G*_ = 0.5 * the scale value of the point of interest, resulting in the following expression:

$$threshold=\bar{\theta (x,y)}*0.8$$ (8)

$$Gussian\;scale\;value=(\theta (x,y)\geq threshold)*\sigma_{G}$$ (9)

With the simplified characteristic vector, the process is continued with the development of the classifier. The main criterion to consider is that the chosen classifier contains the necessary parameters for the classification of the dorsal fin, which must be fast, simple and portable.

Step 4: Classification (Training). We divide this stage into different subsets of steps to improve the analysis performance of all images that are contained in the dorsal fin image database.

Step 4.1. A group of images is randomly selected from each class for the proposed training according to the classification made by the CICIMAR-IPN researchers [33,48]. We worked according to these classes, which are right falcate, left falcate, right hook, left hook, right triangular and left triangular and selected 20 images from each class for the training.

Step 4.2. For the training system, the weights are added to the descriptors of each class in the training samples, as shown in Eq 10.

$$median\;value(j,x,y)=\frac{\sum_{j=1}^{class\;number}\sum_{x=1}^{N}\sum_{y=1}^{N}z(x,y,j))}{class\;number}$$ (10)

where z is the value of the contrast change of the dorsal fin contour image, N is the number of samples taken by each class, and j corresponds to each of the classes used for classification.

Step 4.3. For each class, the number of matching points between the sample image (original sample after ROI selection) and the samples used in the training must be obtained.

The number of intersections is calculated with the norm of the Euclidean distance between two samples and the ordinate with the minimum distance between them.

$$descriptor\;distance=\sum_{j=1}^{c1ass\;number}\sum_{x=1}^{N}\sum_{y=1}^{N}∥L(j,x,y,\sigma )-median\;value(j,x,y){∥}_{L2}^{2}$$ (11)

where the class number is the total number of classes to classify, N is the number of training samples, x and y are the coordinates of the vector at the current training position, *L (j*, *x*, *y*, *σ)* is the position of the value in the vector of the image at different scales, and ∥∥_*L*2_ is the norm of vector L2.

**Fig 5:**
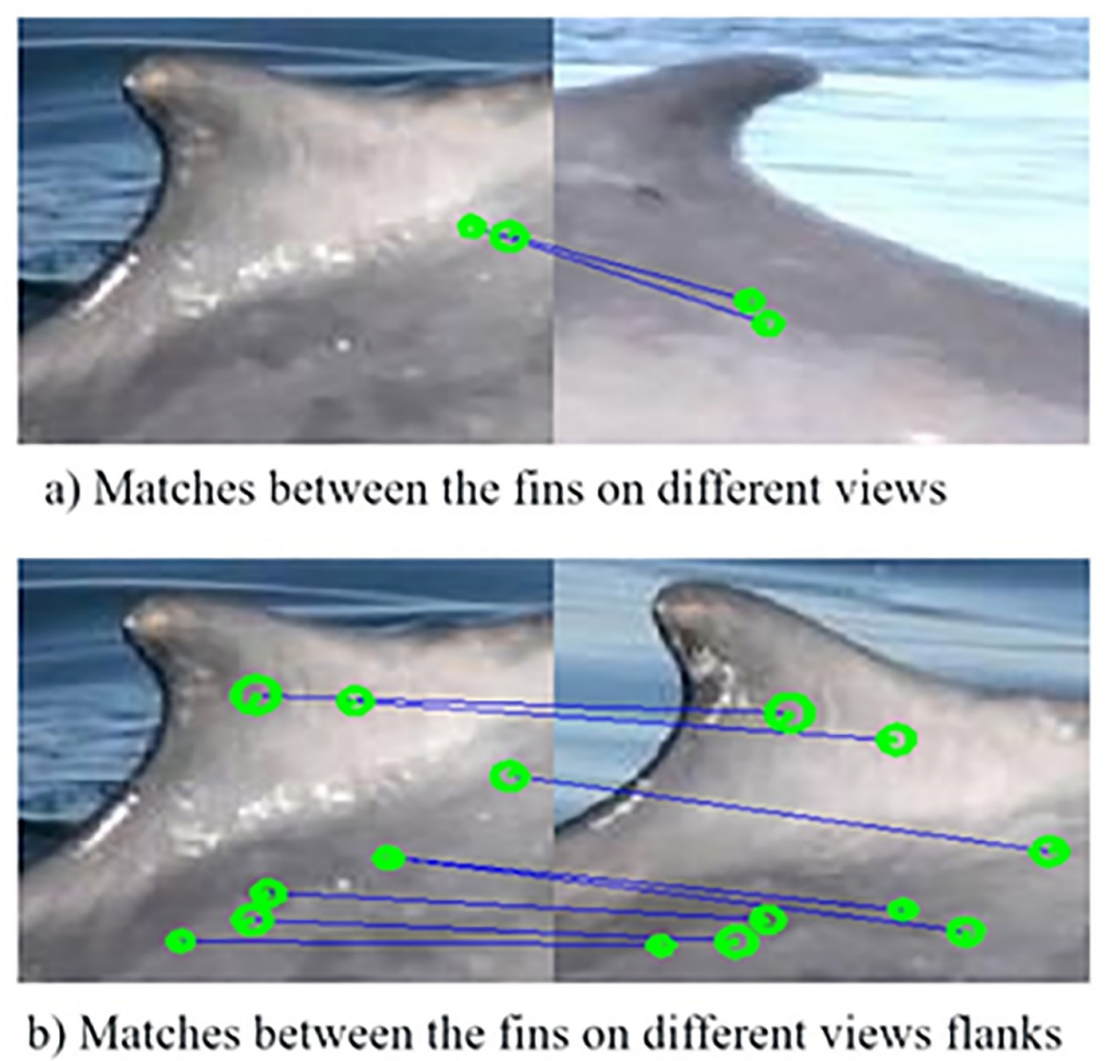
Distance between the characteristic points obtained from Eq 11.

A graphical interpretation of the method is shown in Fig 5A and 5B, where the correspondence between each of the selection points on the blue whale dorsal fin can be observed.

Additionally, when we developed the test scenarios, four metrics were considered: true positive (TP) and true negative (TN)—which are for correct classifications—and false positive (FP) and false negative (FN)—which are for incorrect classifications; by using these metrics, we can obtain different performance measures as follows [46]:

$$Sp=\frac{TN}{\left(TN+FP\right)}$$ (12)

$$Se=\frac{TP}{\left(TP+FN\right)}$$ (13)

$$Acc=\frac{\left(TP+TN\right)}{pixels\;of\;cetacean\;image}$$ (14)

Specificity (Sp) is the ability to detect nonblue whale pixels, sensitivity (Se) reflects the algorithm's ability to detect the edge of the blue whale dorsal fin, and accuracy (Acc) measures the proportion of the total number of pixels correctly classified (sum of the true positives and true negatives) to the total number of pixels that correspond to the image of the whale [46]; the accuracy is the probability that a pixel belonging to the whale is correctly identified.

### Classifier presentation on the Jetson Tegra TK1

**Fig 6:**
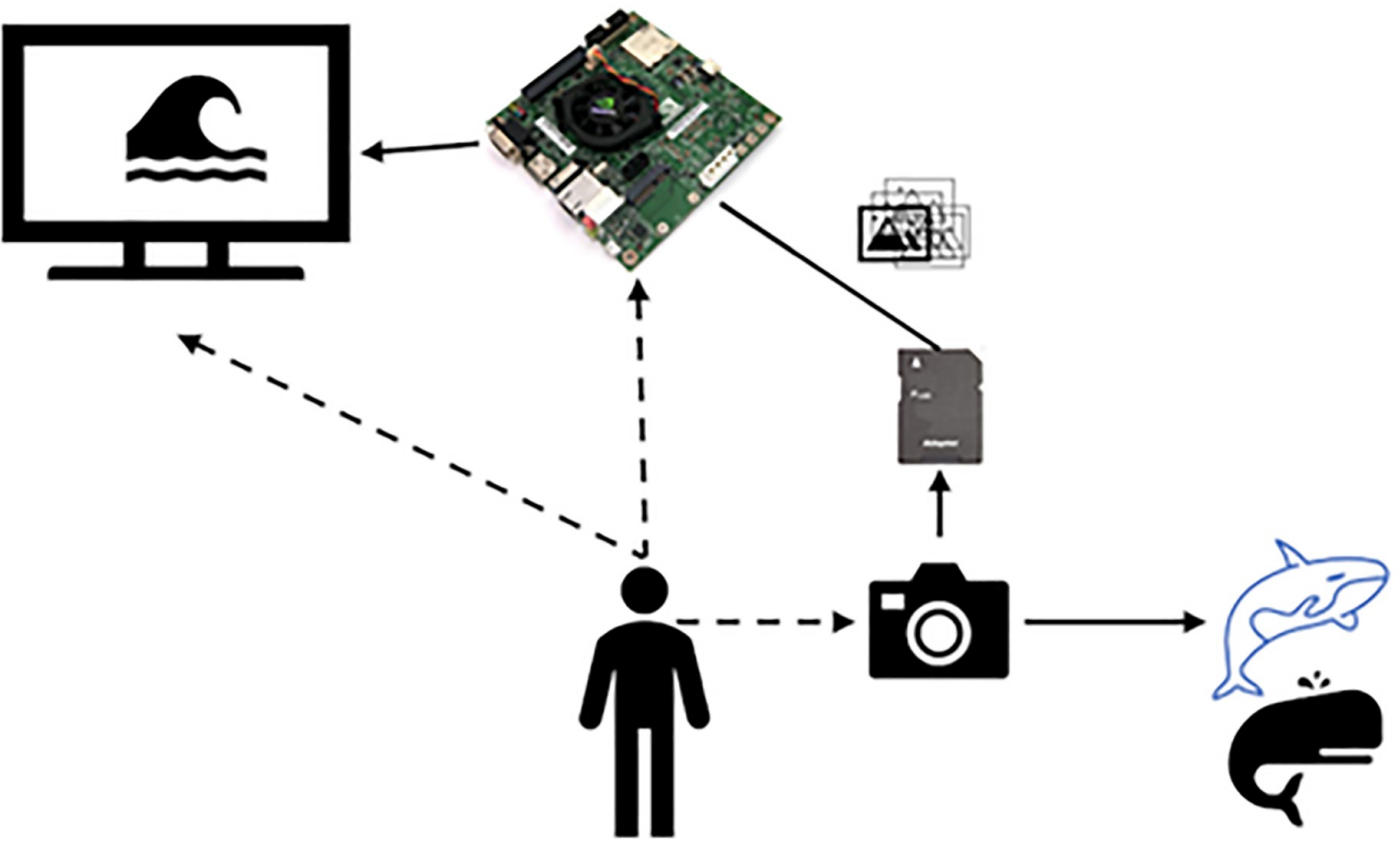
Blue whale photo-id process.

This section briefly shows the architecture of the blue whale photo-id process, as shown in Fig 6.

**Fig 7:**
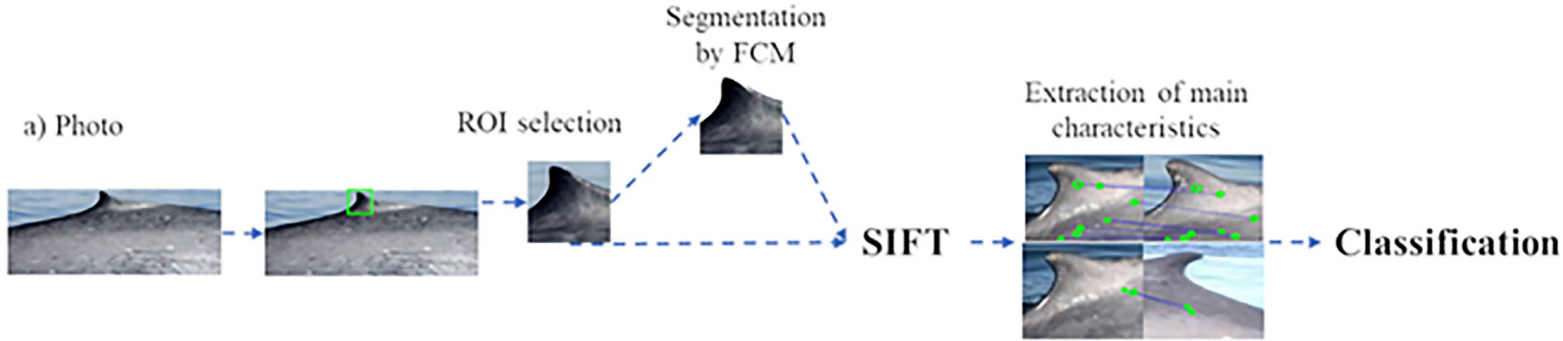
General photo-id whale process.

**Fig 8:**
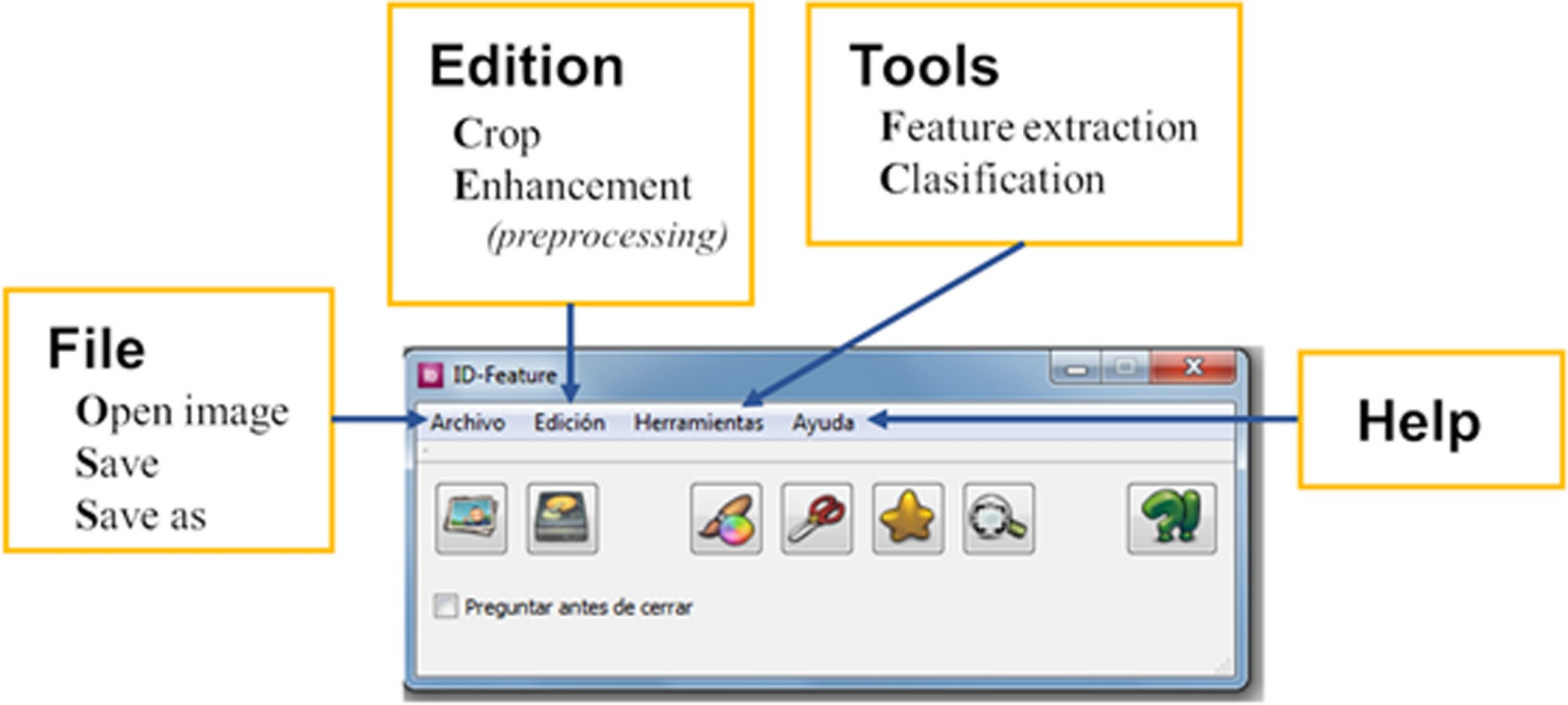
Graphic presentation of photo-id whale.

Fig 6 shows the following process. i) The user takes an image with a camera. ii) The collected images are stored in memory, which is located on the Jetson Tegra TK1 mobile platform, where the identification and classification process developed in this work is performed, as shown in Fig 7.

iii) Presentation of Photo-Id Whale results. To carry out the photo-id of the blue whale, the elements of the process were developed in a concise and brief way: upload a photo, select the ROI, and obtain and store information. These elements are as shown in Fig 8.

**Fig 9:**
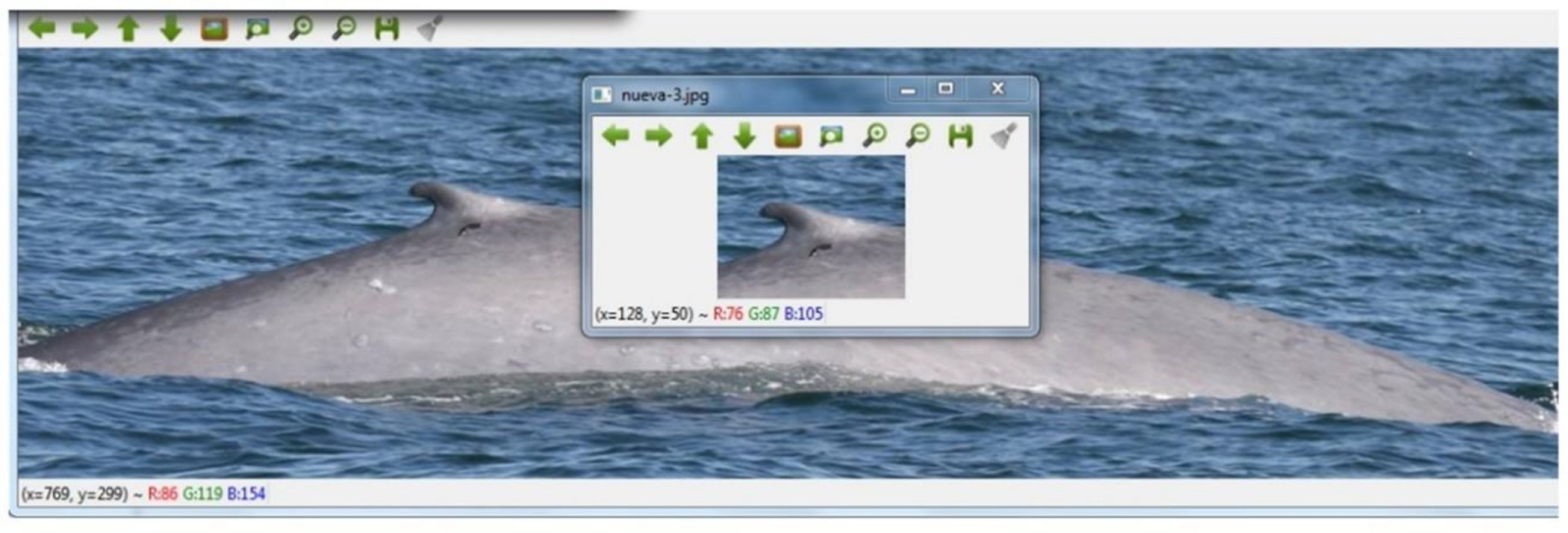
Photo-id whale final result.

Fig 8 shows the options for identifying and storing the dorsal fin image of a blue whale. The File menu has the following options: open image, save and save as. Next, there is the editing menu with the following options: cut and improvement (preprocessing). Later, the tools menu has the following options: extraction of characteristics and classification. Finally, the help menu consists of a brief explanation of the development. The final result is shown below Fig 9.

https://doi.org/10.17504/protocols.io.be2rjgd6

## Results

The classification tests were run using the CICIMAR-IPN blue whale database, which is a collection of 1172 images, where 697 show a falcate fin contour, 326 show a hooked fin contour and 149 show a triangular fin contour. Among the photographs in the blue whale image database, 57.2% were taken from both sides of the whale, 23.8% were taken from the right flank and 19.0% were taken from the left flank. To validate the proposed statistical classifier, tests were performed with segmented images (SI) and unsegmented images (UI) of the contour of the blue whale dorsal fin. To evaluate the performance related to the processing time (PT) of the proposed methodology, we tested the algorithms on a laptop computer with an Intel (R) Core (TM) i7-2630QM CPU @ 2.00 GHz with 6 GB of RAM and a 64-bit operating system and the image processing tests were also carried out on a Jetson Tegra TK1 mobile platform.

### Performance of the proposed methodology on images with segmented ROIs

Tables 1–3 show the performance results obtained for the proposed classifier based on the reduced data extracted from triangular, hooked and falcate dorsal fins in terms of the Sp, Se, and Acc; each of the performance tests were performed on the Jetson Tegra TK1. To evaluate the performance of the statistical classifier, four different training processes were performed. To train this classifier, 300 images were randomly selected. These 300 image samples were divided into six classes, corresponding to the different shapes of the blue whale dorsal fin: right falcate, left falcate, right hooked, left hooked, right triangular and left triangular.

**Table 1:** Performance results of the median estimation classifier on images with segmented ROIs.

Statistic	Right Falcate	Left Falcate	Right Hooked	Left Hooked	Right Triangular	Left Triangular
**Sp (%)**	85	98	87	86	93	95
**Se (%)**	70	40	64	77	86	28
**Acc (%)**	82	79	83	86	93	84

**Table 2:** Performance results of the median estimation classifier on images with unsegmented ROIs.

Statistic	Right Falcate	Left Falcate	Right Hooked	Left Hooked	Right Triangular	Left Triangular
**Sp (%)**	74.62	77.33	91.31	92.78	95.37	95.98
**Se (%)**	58.41	50.63	39.26	24.92	95.23	41.42
**Acc (%)**	72.68	72.32	81.23	66.27	95.36	91.44

**Table 3:** Comparative performance of the Acc of different classifiers in classifying dorsal fin images of the blue whale.

	Standard deviation of the Acc obtained by different feature extraction methods		
Classifier	HU invariant moments	SIFT	Acc ≥ 79%
**Minimum distance**	0.0476		
**K-NN, with K = 3**	0.0574		
**K-NN, with K = 5**	0.0496		
**K-NN, with K = 7**	0.0385		
**K-NN, with K = 9**	0.0446		
**Median estimation with segmented ROI**	---------	0.0476	0.0476
**Median estimation with unsegmented ROI**	---------	0.1157	0.1157

### Performance of the proposed method on images with segmented ROIs

The results of the three tests for the proposed method are shown in Table 1. The best result with respect to the Acc is obtained with the right triangular fin class with a value of 93% while the left falcate fin class had the lowest Acc at 79%. In the Se test, the best performance was found with the right triangular fin class at 86%, and the lowest was found with the left falcate fin class at 40%. Finally, for the Sp test, the best result was obtained with the left falcate fin class at 98%, and the lowest result was obtained with the right falcate fin class at 85%.

**Fig 10:**
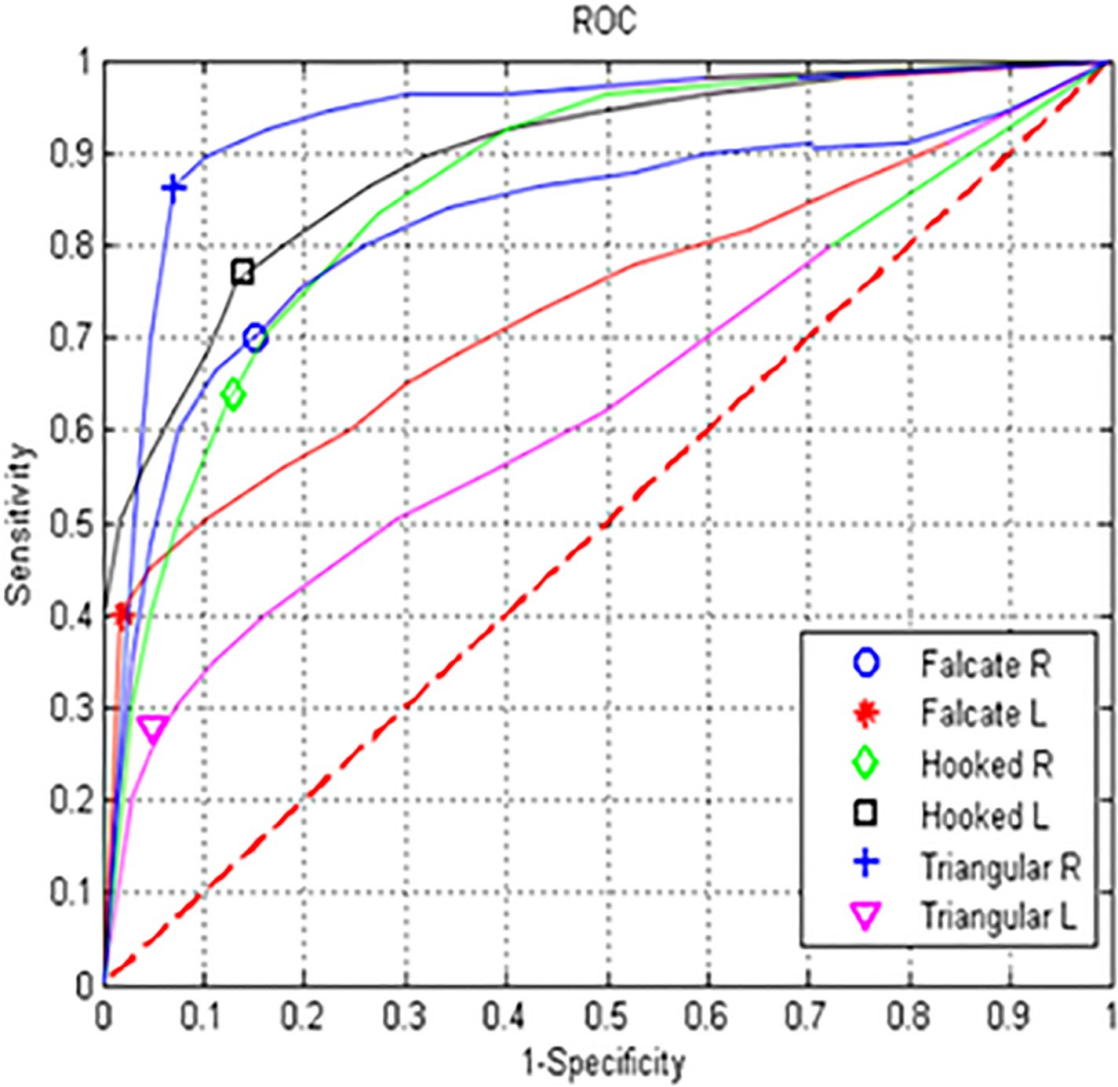
ROC curves for experiment # 1.

Fig 10 shows the results obtained from the proposed method for manually segmented images in terms of the ROC curves, where the right triangular fin class shows the best performance. The lowest performing classes were the left triangular fin and the left falcate fin classes.

### Performance of the proposed method on images with unsegmented ROIs

Table 2 shows the results from this experiment, which demonstrates that the best Acc was obtained for the right triangular fin class at 95.36% and the lowest was obtained for the left hooked dorsal fin class at 66.27%. In terms of the Se, the best performance was obtained for the right triangular fin class at 95.23%, and the lowest was obtained for the left hooked fin at 24.92%. Finally, for the Sp, the best performance was obtained for the left triangular fin class at 95.98%, and the lowest was obtained for the right falcate fin class at 74.62%.

**Fig 11:**
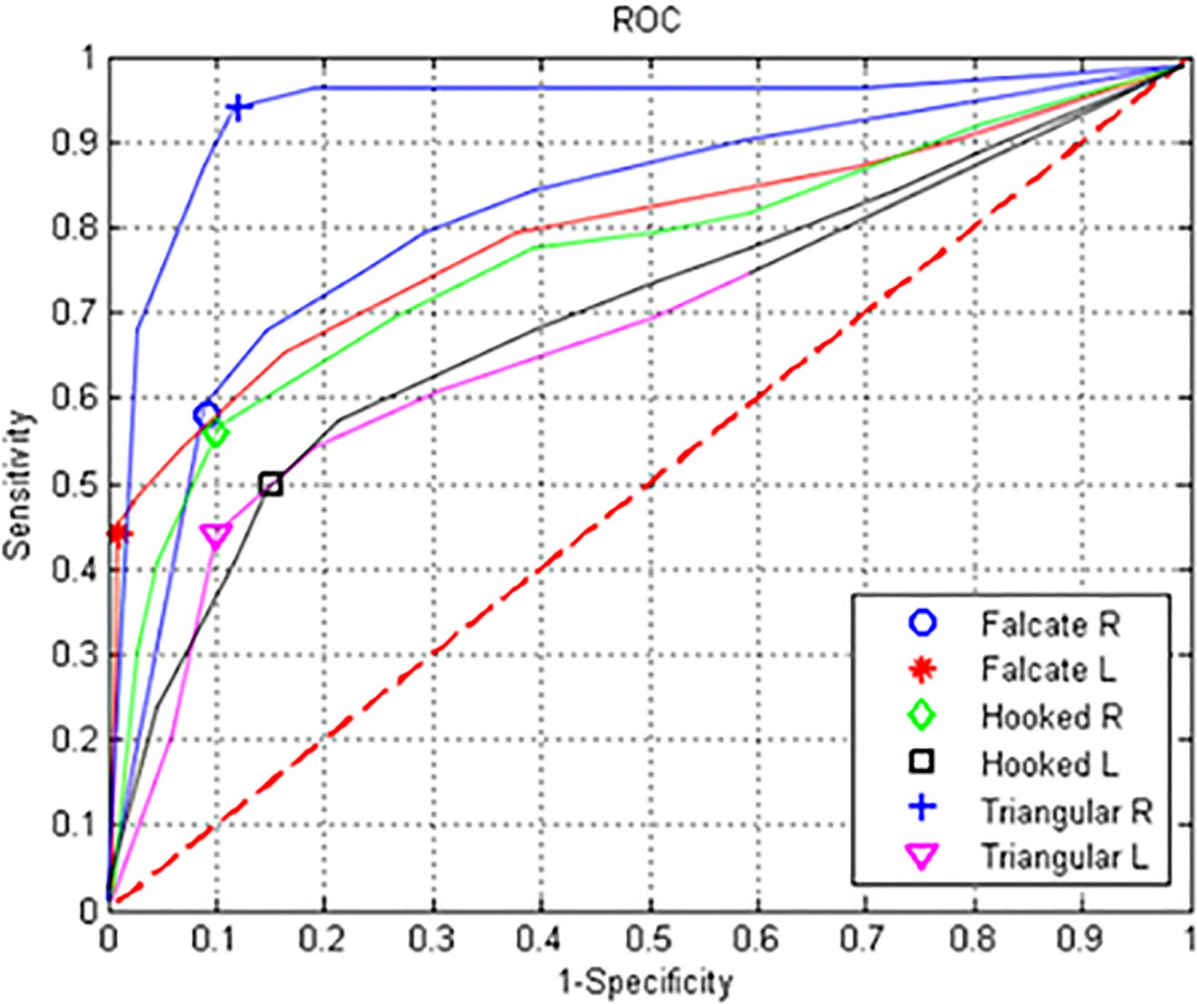
ROC curves for experiment #2.

Fig 11 shows the ROC curves, visually illustrating the results of the proposed method for unsegmented images of the dorsal fin and demonstrating that the right triangular fin class had the best performance while the left and right hooked fin classes had the lowest performance.

To validate this investigation, the proposed classifier (the Median Estimation Classifier) was compared with other classifiers. We analyzed the standard deviation of the accuracy for the six fin classes (right falcate, left falcate, right hook, left hook, right triangular and left triangular) in the experiments developed for the identification of dorsal fins. Table 3 shows the results obtained by applying the standard deviation of the Acc metric, which are calculated for each comparison between the proposed classifier and the different, existing classifiers. We can see that the classifier with the lowest standard deviation was the K-NN classifier with K = 7 (0.0385), but the Acc was less than 79% for the following classes: left falcate fin (75.83%), right hooked fin (73.33%), and left hooked fin (78.33%). The K-NN classifier for K = 9 yielded similar Acc values, with 75.85% for the left falcate fin class and 74.16% for the right hooked fin class. The opposite result is shown for the same classifier in experiment 2 (without image segmentation), where a larger standard deviation was obtaining for each of the Acc values. The Acc values obtained were below 79% for the following classes: right falcate fin (72.68%), left falcate fin (72.32%) and left hooked fin (66.27%).

### Performance processing time of the proposed method

Tables 4 and 5 respectively show the results for all six classes of fins with and without segmentation, including the average performance in terms of the processing time (PT) given in seconds with the Jetson Tegra TK1mobile platform compact GPU hardware and the Intel i7 CPU laptop computer.

**Table 4:** Runtimes for different images with and without segmentation (proposed classifier).

Equipment	RF (s)	RF (s)	LF (s)	LF (s)	RH (s)	RH (s)	LH (s)	LH (s)	RT (s)	RT (s)	LT (s)	LT (s)
	ROI/NS	ROI/S	ROI/NS	ROI/S	ROI/NS	ROI/S	ROI/NS	ROI/S	ROI/NS	ROI/S	ROI/NS	ROI/S
**Laptop computer**	2.54	1.85	2.56	1.53	2.53	1.91	2.57	1.54	2.77	2.13	2.62	1.77
**Laptop computer**	2.56	1.86	2.54	1.58	2.49	1.90	2.53	1.52	2.79	2.13	2.69	1.87
**Laptop computer**	2.54	1.86	2.57	1.59	2.52	1.89	2.55	1.55	2.77	2.15	2.55	1.79
**Laptop computer**	2.59	1.86	2.52	1.61	2.47	1.94	2.55	1.50	2.93	2.14	2.54	1.77
**Laptop computer**	2.54	1.84	2.55	1.53	2.59	1.89	2.51	1.53	2.75	2.16	2.57	1.82
**Average PT**	**3.09**	**1.85**	**2.54**	**1.56**	**2.52**	**1.90**	**2.54**	**1.52**	**2.80**	**2.14**	**2.59**	**1.80**

**Table 5:** Runtimes for the different images with and without segmentation (proposed classifier).

Equipment	RF (s)	RF (s)	LF (s)	LF (s)	RH (s)	RH (s)	LH (s)	LH (s)	RT (s)	RT (s)	LT (s)	LT (s)
	ROI/NS	ROI/S	ROI/NS	ROI/S	ROI/NS	ROI/S	ROI/NS	ROI/S	ROI/NS	ROI/S	ROI/NS	ROI/S
**Jetson Tegra TK1**	1.49	1.11	1.44	0.99	1.24	1.20	1.40	1.15	1.21	0.84	1.59	0.92
**Jetson Tegra TK1**	1.40	1.10	1.47	0.80	1.57	1.11	1.42	1.19	1.24	0.75	1.54	1.36
**Jetson Tegra TK1**	1.50	1.46	1.48	0.99	1.40	1.15	1.47	0.91	1.47	0.83	1.46	0.87
**Jetson Tegra TK1**	1.30	1.11	1.45	1.10	1.47	1.36	1.45	1.36	1.50	0.96	1.46	0.98
**Jetson Tegra TK1**	1.38	1.19	1.56	0.95	1.92	0.97	1.46	1.11	1.41	0.95	1.33	0.97
**Average PT**	**1.41**	**1.19**	**1.48**	**0.96**	**1.52**	**1.15**	**1.44**	**1.14**	**1.36**	**0.86**	**1.47**	**1.02**

The results in Tables 4 and 5 show that the best processing time of 0.9 seconds was obtained for the left and right triangular fin classes, particularly for the images with segmented ROIs.

## Discussion

Photo-id systems have been developed for humpback whales, bottlenose dolphins, manta rays, etc. Currently, however, there is no system dedicated to the photo-id of the blue whale fin contours, which is more reliable than identification using the skin pigmentation in the blue whale since the latter can be affected by skin flaking caused by dietary changes, temperature, and the salinity of the sea. For this reason, the results obtained by the PhotoId-Whale classifier were compared with those obtained with the method in [49], which performs the photo-id of the humpback whale, the sperm whale and the pilot whale. The method presented in [50] uses a chain code, which consists of obtaining a border pattern for each interval when there is a change in the direction of the border. This method was implemented for the identification of the fin shape of dolphins and whales, although the runtime results were not shown due to the nature of their algorithm, which is slow and consumes a large amount of computing resources [51]. The reason for the high computational costs of the chain code is the greater image quality due to the better resolution of images obtained today compared with the images taken with professional cameras 10 years ago. As a result of this technological advance, clearer details can be seen in the images of the edges and contours of the dorsal fin of the blue whale. In this work, we carried out the search and identification of the blue whale in dorsal fin images from a blue whale dorsal fin database through the Jetson Tegra TK1, which meets the requirements of low energy consumption and can simultaneously compare newly acquired images of the dorsal fin taken from the right and left flanks to those in the different classes described earlier in the document.

The results obtained for Experiment #1 showed that the triangular fin class had the highest Acc at 93% while the left falcate fin class had the lowest Acc at 79%. In the Se test, the best performance was obtained for the right triangular fin class with 86%, while the lowest was obtained for the left falcate fin class at 40%. Finally, for the Sp test, the best performance was obtained for the left Falcate fin class at 98%, and the lowest was obtained for the right falcate fin class at 85%.

For Experiment #2, the best Acc was obtained for the right triangular fin class at 95.36%, and the lowest was obtained for the left hooked dorsal fin class at 66.27%. In terms of Se, the best performance was obtained for the right triangular fin class at 95.23%, and the lowest was obtained for the left hooked fin class at 24.92%. Finally, for Sp, the best performance was obtained for the left triangular fin class at 95.98%, and the lowest was obtained for the right falcate fin class at 74.62%. Across both experiments, a PT of 0.9 seconds was obtained for the identification of the dorsal fin of the blue whale, far outperforming other photographic identification methods such as Europhlukes (EC EuroPhlukes Initiative, University of Leiden, The Netherlands), which had a PT of more than 90 seconds. Unlike previous methods, the PhotoId-Whale determines and catalogs the blue whale dorsal fin type on a mobile platform, which provides independence for monitoring the blue whales off the Mexican coast. The results obtained in this study demonstrates that the reduction of the data of the characteristic vectors obtained from SIFT in conjunction with the implemented statistical estimator provides accurate and timely results for the identification of the dorsal fin of the blue whale compared to the computational costs shown in the works of [34,35]. In addition, it was found that this method is portable since it was developed in the field through mobile technologies, offering superior performance unlike other development boards, as shown in [51]. One of the advantages of PhotoId-Whale is that the results are obtained using images acquired with standard cameras that do not contain infrared light, irrespective of the incident light, the movement of objects, the time of day or the presence of background objects, which decreases the observation and monitoring costs. The proposed methodology is simple and uses basic algorithms such as SIFT, mathematical expectations, standard deviations, and characteristic vector thresholding, unlike more complex systems that require high calculation costs, such as 13S [31]. The lower computational costs of PhotoId-Whale make the field identification and classification of blue whale dorsal fin images in field research possible, and it is available to scientists and users interested in participating in conservation and ecology activities. Technological developments of this type allow interoperability, maximizing the use and management of information in an accessible and simple way, and improving the user experience that was previously developed using a manual process. The interoperability of this development, the decreased computational costs, and the optimization of the SIFT algorithm allow for the method to be feasibly installed on smartphones, tablets and notebooks. The results show that the best processing time of 0.9 seconds was obtained for the left and right triangular fin classes, particularly for the images with segmented ROIs.

## Conclusions

By acquiring characteristic vectors from SIFT, the number of samples required to obtain the threshold by means of the variance, the mean and the standard deviation is reduced. The results were obtained for segmented and unsegmented ROIs from the images of the dorsal fin of the blue whale by means of two comparative classifiers with the best performance obtained based on the proposed statistical estimation method for sample reduction. The experimental results reveal that the proposed method achieves better performance in terms of the Acc and computational costs (time) in most cases compared to the existing results. We can also conclude that the proposed algorithm is easy to operate in terms of the preprocessing of the images obtained in the blue whale habitat and that the implemented application is feasible on mobile devices, despite their battery and memory limitations.

The results were also validated by plotting the ROC curves and obtaining the AUC values of the curves, which showed that the automatic photographic identification system provides positive results and, therefore, supports the photographic identification of the blue whale in its natural habitat. Finally, in this work, the optimization of the SIFT algorithm is presented to reduce the redundant data. The results validate that the said optimization obtains relevant results focused on the energy consumption, processing time, and preserving the result-processing time relationship. This improvement can be validated on devices with limited resources such as tablets or mobile platforms. The method provides a conservation and ecology tool that may be used by scientists and the general public.
